# DNA interference by a mesophilic Argonaute protein, CbcAgo

**DOI:** 10.12688/f1000research.18445.2

**Published:** 2020-01-09

**Authors:** Nieves García-Quintans, Laurie Bowden, José Berenguer, Mario Mencía

**Affiliations:** 1 Centro de Biología Molecular Severo Ochoa, Universidad Autónoma de Madrid-Consejo Superior de Investigaciones Científicas, Madrid, Madrid, 28049, Spain

**Keywords:** Argonaute, prokaryotic, mesophilic, gene edition, characterization, DNA-DNA interference

## Abstract

**Background**: The search for putative enzymes that can facilitate gene editing has recently focused its attention on Argonaute proteins from prokaryotes (pAgos). Though they are structural homologues of human Argonaute protein, which uses RNA guides to interfere with RNA targets, pAgos use ssDNA guides to identify and, in many cases, cut a complementary DNA target. Thermophilic pAgos from *Thermus thermophilus*, *Pyrococcus furiosus* and *Methanocaldococcus jasmanii* have been identified and thoroughly studied, but their thermoactivity makes them of little use in mesophilic systems such as mammalian cells.

**Methods**: Here we search for and identify CbcAgo, a prokaryotic Argonaute protein from a mesophilic bacterium, and characterize *in vitro* its DNA interference activity.

**Results**: CbcAgo efficiently uses 5’P-ssDNA guides as small as 11-mers to cut ssDNA targets, requires divalent cations (preferentially, Mn ^2+^) and has a maximum activity between 37 and 42 °C, remaining active up to 55 °C. Nicking activity on supercoiled dsDNA was shown. However, no efficient double-strand breaking activity could be demonstrated.

**Conclusions**: CbcAgo can use gDNA guides as small as 11 nucleotides long to cut complementary ssDNA targets at 37ºC, making it a promising starting point for the development of new gene editing tools  for mammalian cells.

## Introduction

Argonaute proteins play a central role in gene silencing and defense against external RNA in eukaryotes, binding small RNA molecules that are then used as guides to scan for complementary RNA targets in the form of mRNAs or RNA viruses. Depending on the presence of specific residues in the protein sequence, the targets can be cut or simply blocked, with degradation carried out by other proteins, leading to inhibition of the expression (silencing) of the genes involved ^1^. The structure of eukaryotic Argonaute proteins (eAgo) consists of four domains organized in a specific order (N-PAZ-MID-PIWI) which are each involved in different steps of the protein’s enzyme activity.

Homologues to eAgo that contain these four domains are found in both bacteria and archaea, being collectively known as prokaryotic Argonaute (pAgo) proteins. Their function seems to depend on the species, targeting either RNA or DNA ^2^. The best studied pAgos, such as those from *Thermus thermophilus* (ThAgo) ^3^, *Pyrococcus furiosus* (PfAgo) ^4^ and *Methanocaldococcus jannaschii* (MjAgo) ^5^, use ssDNA guides (gDNA) to target DNA *in vitro,* with ThAgo and PfAgo shown to be involved in defense against invading DNA *in vivo* ^3, 4^. In contrast, pAgo from *Aquifex aeolicus* (AeAgo) ^6^ and *Natronobacterium gregoryi* (NgAgo) ^7^ use ssDNA guides to target RNA, suggesting a putative role in gene silencing, similar to that of eAgo, or in defense against RNA viruses. Moreover, other pAgos, like that of *Rhodobacter spheroides* (RsAgo), use RNA guides against DNA targets, maintaining its defense capability against invading DNA despite the absence of endonucleolytic activity in its PIWI domain ^8^.

High-resolution structures of pAgos in complex with guide and target DNAs support a mechanism of hydrolysis homologous to that of RNAse H, in which an Asp-Glu-Asp-Asp catalytic tetrad is formed at the cleavage site of its PIWI domain upon scanning and hybridization of gDNA and target ssDNA ^9^. However, the actual mechanism for generation of the gDNA *in vivo* is essentially unknown and the described *in vitro* capability of the MjAgo ^5^ and ThAgo ^10^ apoproteins to cleave dsDNA (named DNA chopping) seems an unlikely mechanism for the generation of gDNA *in vivo*, as it cannot explain the observed inactivity against its own genomic DNA.

Following description of the mechanism of action of ThAgo and PfAgo, the possibility of using pAgos as tools for gene editing has been proposed, with the advantage of being easier to use than the CRISPR-Cas9 system ^11^. However, attempts to directly use ThAgo for gene editing of mammalian cells were unsuccessful, likely due to the thermoactivity of this protein (unpublished results of our laboratory). Publication of gene editing of mammalian cells using NgAgo, a mesophilic pAgo from a hyperhalophilic archaea ^12^, sparked controversy due to the inability of many other laboratories to reproduce the results ^13^. Other published research has suggested that the substrates for NgAgo are RNA targets ^7^. Despite this, the search has continued for new pAgos that could be successful in gene editing at low temperatures through a DNA-DNA interference mechanism ^11^.

At the time of writing this article, an unreviewed preprint article describing the properties and structure of a mesophilic pAgo derived from *Clostridium butyricum* (CbAgo) was posted online by the group of John van der Oost, immediately followed by an article of the group of Alexei A. Aravind describing biochemically the same CbAgo protein and also an pAgo protein from *Limnothrix rosea* (LrAgo). Both articles were further published ^14, 15^. Here we show our independent and contemporary work leading to the identification of a pAgo from the strain CWBI 1009 of *C. butyricum* (CbcAgo thereafter), describing its properties in comparison to that of the CbAgo protein characterized in these articles, including a requirement for 5’-phosphorylated gDNA and a smaller minimum gDNA size required for full activity.

## Methods

### Identification, overproduction and purification of CbcAgo

The search for mesophilic pAgos was performed using the web interface of the BLASTp program, with the protein sequence of *Natronobacterium gregoryi* (WP_005580376.1) as a query, and directed to non-redundant GenBank CDS translations + PDB + SwissProt + PIR + PRF, excluding environmental samples from WGS projects. Using the default settings, proteins from two strains of *Clostridium butyricum* were identified (WP_045143632.1 and WP_058142162.1). Further BLASTp and COBALT analysis also with default settings revealed the presence of the four domains that characterize pAgos and the residues required for their likely nuclease activity within the PIWI domain. A fusion gene encoding an N-terminal Strep (II) tag and protein WP_045143632.1 from the strain *C. butyricum* CWBI1009 (CbcAgo) was synthesized (GenScript) following the codon usage of *E. coli* and cloned into a pET11d vector (Agilent Technologies) to generate the expression plasmid pET11-CbcAgo. For overexpression in *E. coli* KRX strain (genotype [F´, *tra*D36, *Δomp*P, *pro*A ^+^B ^+^, lacI ^q^, Δ( *lac*Z)M15] Δ *omp*T, *end*A1, *rec*A1, *gyr*A96 (Nal ^r^), thi-1, hsdR17(r _k_^-^, m _k_^+^), e14 ^-^ (McrA ^-^), *rel*A1, *sup*E44, Δ( *lac-pro*AB), Δ( *rha*BAD)::T7 RNA polymerase) (Promega), cultures were grown at 37 °C in LB with 100 μg/ml ampicillin until an optical density at 600 nm (OD _600_) of 0.7 was reached. CbcAgo expression was induced by the addition of 1 mM IPTG (isopropyl β-D-1-thiogalactopyranoside) and 0.1% (w/v) of L-Rhamnose to the growth medium with further incubation at 20 °C for 18 h with mild shaking. This procedure resulted in the production of a protein of the expected size (90.4 kDa, 794 amino acids), as revealed by SDS-PAGE in comparison with size standards. After lysis by French press in TrisHCl 50mM, 1M NaCl, pH 8 buffer and elimination of insoluble debris by centrifugation (30 min / 30,000 × g / 4 °C) and filtration (Acrodisc Syringe Filter 0.45 μm, Life Sciences), the protein was purified by affinity chromatography in Strep-Tactin sepharose (IBA, Germany, Cat no. 2-1201-010). The CbcAgo protein concentration was measured by comparison with known concentration of BSA in Comassie-Blue stained SDS-PAGE using ImajeJ for quantification. Aliquots were stored at -20 °C with 40% glycerol until use. As a negative control for the interference assays, a double mutant lacking the catalytic tetrad (D541A, E577A) was generated (called DE mutant hereafter) by site-directed mutagenesis, (Quick change II site-directed mutagenesis kit, Agilent Technologies) overexpressed from plasmid pET11-CbcAgoDE and purified in the same way.

Proteins purified by this method were separated in an SDS-PAGE gel, digested with trypsin and chemotrypsin and the resulting peptides were identified by LC-MS/MS in an LTQ Orbitrap Velos Pro (high resolution, short gradient) equipment.

### DNA interference assays

The synthetic gDNA and ssDNA targets described in Table 1 (SIGMA-ALDRICH) were used for the interference assays. The standard interference assays were carried out as follows: after pre-incubation of the CbcAgo protein (6 μM) with a given primer (6 μM), selected among those described in Table 1, for 10 min at 37 °C in reaction buffer (20 mM Tris HCl, 150 mM NaCl, 2 mM of MnCl _2_, pH 7.5). The ssDNA target (1.2 μM) was added and the samples were incubated in the same buffer for 50 min at 37 °C, except when otherwise indicated. After incubation, the reaction was stopped by the addition to the sample of one reaction volume of loading buffer (85% formamide, 10 mM EDTA, 20% glycerol, 0.05% bromophenol blue, 0.05% xylene cyanol) and further heating for 10 min at 100 °C. Separation of the ssDNA substrate, products and gDNA was carried out by electrophoresis in an 18–20% polyacrylamide gel in the presence of 6M urea (U-PAGE), using SYBR Gold Nucleic Acid Gel Stain for staining (Invitrogen S11494) and a UV spectrophotometer for detection. Synthetic oligonucleotides of different sizes were used as mobility standards.

**Table 1.** Sequence of ssDNA targets and guide DNAs used in this work. Table shows the names and sequence of the two target DNA used in this work (T-45 and T-50) and the oligonucleotides used as gDNA. All the gDNA except for 21-OH and 20-OH are phosphorylated at their 5’ end, being labelled as [phos].

Name	Sequence (5´-3´)	Description
T-45	AAACGACGGCCAGTGCCAAGCTTACTATACAACCTACTACCTCAT	DNA target
T-50	GGTCGCGGAGGTTATGGATGCGATCGCTGCGGCCGATCTTAGCCAGACGA	DNA target
W-1	[phos]-TGAGGTAGTAGGTTGTATAGT	DNA guide to 1 nt of 3'-target
W-2	[phos]-GAGGTAGTAGGTTGTATAGTA	DNA guide to 2 nt of 3'-target
W-3	[phos]-AGGTAGTAGGTTGTATAGTAA	DNA guide to 3 nt of 3'-target
W-4	[phos]-GGTAGTAGGTTGTATAGTAAG	DNA guide to 4 nt of 3'-target
W-5	[phos]-GTAGTAGGTTGTATAGTAAGC	DNA guide to 5 nt of 3'-target
W-6	[phos]-TAGTAGGTTGTATAGTAAGCT	DNA guide to 6 nt of 3'-target
W-7	[phos]-AGTAGGTTGTATAGTAAGCTT	DNA guide to 7 nt of 3'-target
W-8	[phos]-GTAGGTTGTATAGTAAGCTTG	DNA guide to 8 nt of 3'-target
20-P	[phos]GAGGTAGTAGGTTGTATAGT	DNA guide to 2 nt of 3'-target
21-OH	TGAGGTAGTAGGTTGTATAGT	DNA guide to 1 nt of 3'-target
20-OH	GAGGTAGTAGGTTGTATAGT	DNA guide to 2 nt of 3'-target
Hy 10-7	[phos]CGTCTGG	7 nt DNA guide
Hy10-9	[phos]CGTCTGGCT	9 nt DNA guide
Hy10-11	[phos]CGTCTGGCTAA	11 nt DNA guide
Hy10-13	[phos]CGTCTGGCTAAGA	13 nt DNA guide
Hy10-15	[phos]CGTCTGGCTAAGATC	15 nt DNA guide
Hy10-17	[phos]CGTCTGGCTAAGATCGG	17 nt DNA guide
Hy10-19	[phos]CGTCTGGCTAAGATCGGCC	19 nt DNA guide
Hygro-1	[phos]TGGTCGCGGAGGTTATGGATGCGATCGCT	DNA guide (Fw)
Hygro-2	[phos]TCATCCATAACCTCCGCGACCGGTTGCAG	DNA guide (Rv)
Hygro-3	[phos]GGTCGCGGAGGTTATGGATGCGATCGCT	DNA guide (Fw)
Hygro-4	[phos]CATCCATAACCTCCGCGACCGGTTGCAG	DNA guide (Rv)

Assays of nicking activity on dsDNA were carried out following the above protocol using plasmid pMH184 isolated from *E. coli* cells in its supercoiled form as a target (GenJET Plasmid Miniprep Kit, Thermo scientific, Cat no. K0503). The molar ratio between CbcAgo: guide: dsDNA target was 3 : 6 : 0.0074 (μM). Reactions were stopped by adding 100 μg/mL of Proteinase K (Promega) and the products were separated in agarose gels. As mobility standards, linear and nicked forms of pMH184 were generated by digestion with *EcoRI* (Thermo scientific, FD0275) and *Nt.BspQI* (Biolabs R06445) restriction enzymes, respectively.

## Results

**Figure 1.**
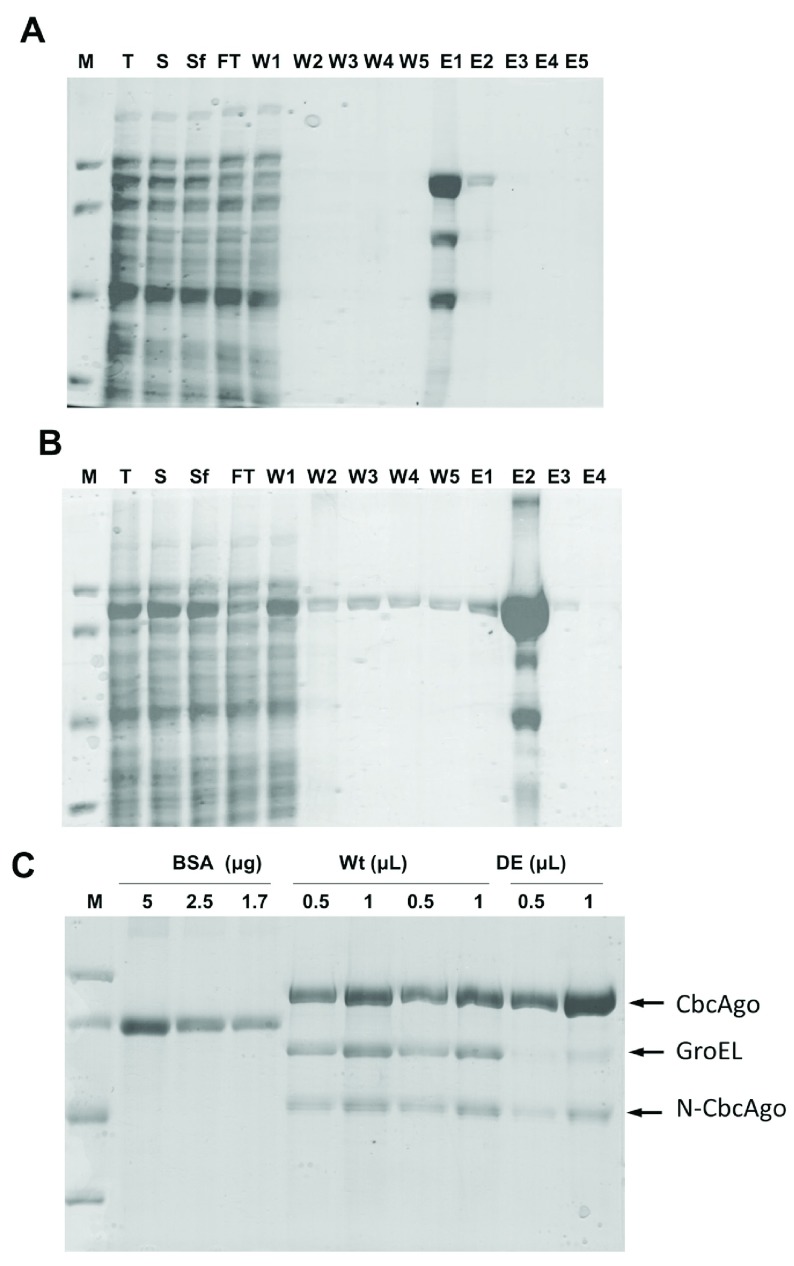
Purification of CbcAgo. Purification by affinity chromatography of ( **A**) wild-type or ( **B**) inactive DE mutant. Lanes: M - molecular weight markers from top to bottom of 97.4, 66.2, 45 and 31 kDa; T - total cell protein fraction; S - soluble fraction; Sf - filtered soluble fraction; FT - flow through; W1-5 - fractions obtained upon addition of washing buffer; E1-5 - fractions obtained upon addition of elution buffer. ( **C**) Protein concentration of two independent preparations of wild-type CbcAgo and a single preparation of the DE mutant, compared with bovine serum albumin as standard (BSA). Protein identification was carried out by proteomic analysis.

**Figure 2.**
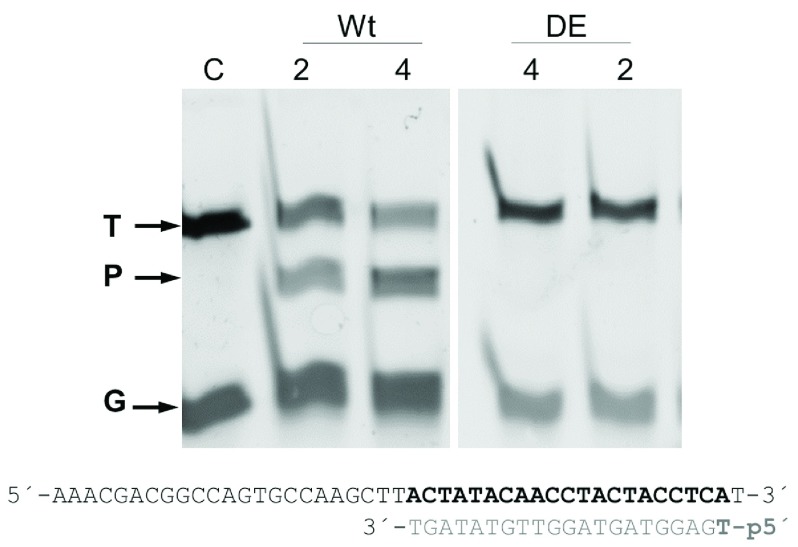
CbcAgo is active in DNA-DNA interference assays. The wild-type (Wt) and inactive DE mutant (DE) of the CbcAgo protein were pre-incubated with the indicated 5’ phosphorylated guide DNA for 10 min at 37 °C and further used to cut a complementary 45-nucleotide ssDNA target at the same temperature for 1 h. The reactions were carried out in the presence of 2 or 4 mM MnCl _2_; target (T), guide (G), and the major 34-mer product (P) of the reaction were identified in an 18% U-PAGE gel.

The Strep II-tagged CbcAgo protein and its inactive DE derivative were overexpressed and purified by affinity chromatography ( Figures 1A and 1B). In addition to the wild-type or DE mutant proteins, two smaller proteins were co-purified at low proportions even after repeated cycles of affinity chromatography. Mass spectrometry of proteolytic Trypsin/Chemotrypsin digestion fragments of these recalcitrant contaminant proteins revealed them to be the GroEL chaperone and an N-terminal fragment of the Strep II-tagged CbcAgo proteins ( Figure 1C). Presence of these co-purified proteins did not interfere with the capacity of the wild-type CbcAgo protein to cleave the ssDNA target after being preloaded with a complementary gDNA guide ( Figure 2). As the DE mutant was inactive in these assays, it was concluded that the endonuclease activity detected in the wild type was dependent on the presence of an active endonucleolytic site in CbcAgo’s C-terminal PIWI domain and was not as a result of hidden activity of the co-purified proteins.

**Figure 3.**
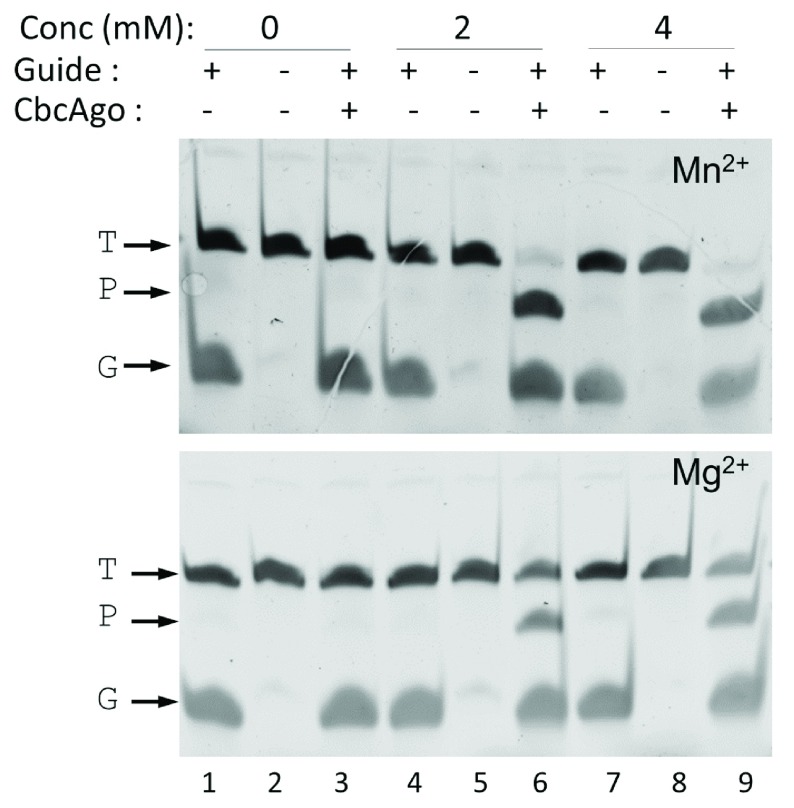
CbcAgo needs divalent cations for activity. CbcAgo was loaded with a 21-mer, 5’-phosphorylated gDNA (G) and incubated with a complementary 45-mer ssDNA target (T) in reaction buffer in the absence (0) or presence of 2 or 4 mM Mn ^2+^ or Mg ^2+^. The major 34-mer product (P) of the reactions was identified in an 18% U-PAGE gel; target and guide were the same used in Figure 2.

**Figure 4.**
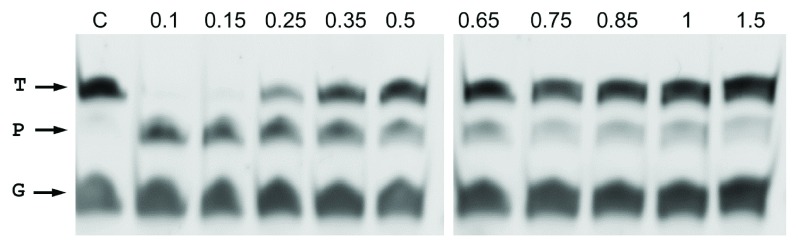
Salt tolerance of CbcAgo. Wild-type CbcAgo was preloaded with the same gDNA used in Figure 2 and incubated with the complementary 45-mer ssDNA target in the presence of the indicated concentrations of NaCl (M). Target (T), guide (G), and the major 34-mer product (P) of the reaction were identified in an 18% U-PAGE gel.

**Figure 5.**
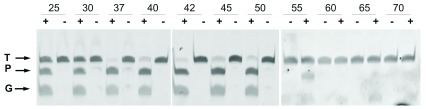
Effect of temperature on CbcAgo. Wild-type CbcAgo was preincubated for 10 min at 37 °C with (+) or without (-) gDNA and then used in cleavage assays of an ssDNA target for 1 h at the indicated temperatures. The ssDNA target (T) and gDNA (G) were the same used in Figure 2.

Optimization of the DNA-DNA interference activity revealed a strict requirement for divalent cations, with higher activity shown in the presence of Mn ^2+^ compared to Mg ^2+^ ( Figure 3), and a significant resistance to high ionic strength ( Figure 4). Subsequent experiments were carried out with Mn ^2+^ and 150 mM of NaCl in the interference buffer. Under these conditions, we then analyzed the range of temperatures at which this endonuclease was active, finding significant activity in the range of 25–55°C, with maximum activity between 37 and 42°C, as revealed by the absence of the target DNA band at this temperature, and no activity at 60°C and above ( Figure 5). All subsequent experiments were carried out at 37°C.

**Figure 6.**
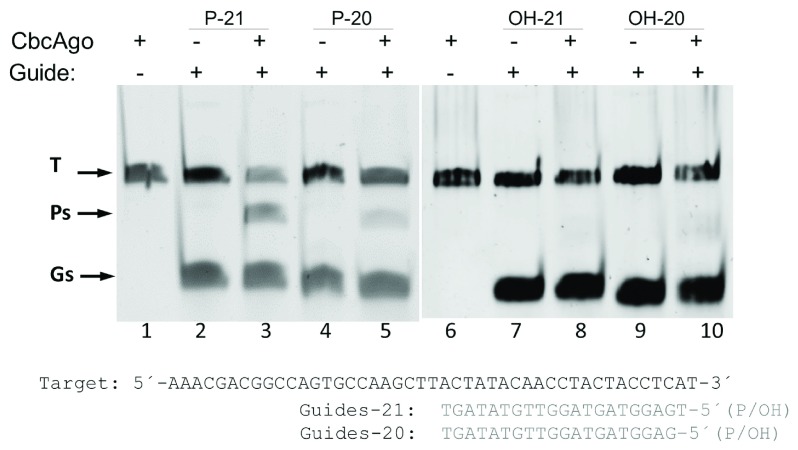
CbcAgo requires 5´-phosphorylated gDNA. The indicated 20- and 21-mer 5’phosphorylated (P) or unphosphorylated (OH) gDNAs were preincubated with CbcAgo and used in interference assays against the same complementary target. Presence (+) or absence (-) of CbcAgo or gDNA in the reaction are indicated. The target (T), guide (G) and the major 34-mer product (P) of the reaction were identified using an 18% U-PAGE gel.

**Figure 7.**
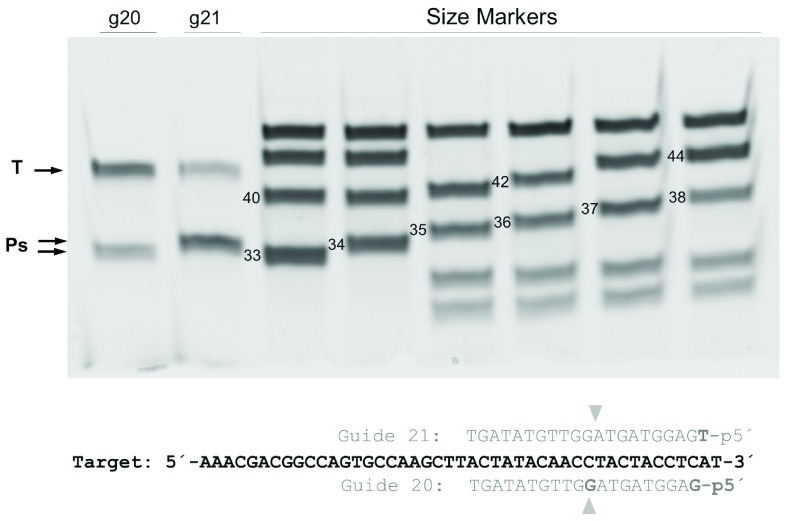
Assessment of the CbcAgo cleavage site. CbcAgo was loaded with 5’-phosphorylated 20- or 21-mer gDNA and used in interference cleavage assays against a 45-mer ssDNA target (T). The sizes of the products (P) were compared with ssDNA standards of the indicated sizes, using a 20% U-PAGE gel, leading to the conclusion that the cleavage site was complementary to the 10–11 base position of the gDNA.

The requirements of the gDNA were also analyzed, clearly showing a need for a 5’ phosphate end, as gDNAs with 5’-OH were unable to direct CbcAgo to the complementary ssDNA target ( Figure 6). The cutting site in the ssDNA target was also analyzed in detail using two gDNAs (20- and 21-mers) with a single nucleotide difference at the 5’-phosphorylated end, comparing the size of the largest product of the reaction with ssDNA size markers. As shown in Figure 7, the products obtained had sizes of 33 and 34 nucleotides for the 20- and 21-mer gDNA respectively. The localized cutting site in the target was complementary to the +10 and +11 position with respect to the 5’ end of the gDNA used.

**Figure 8.**
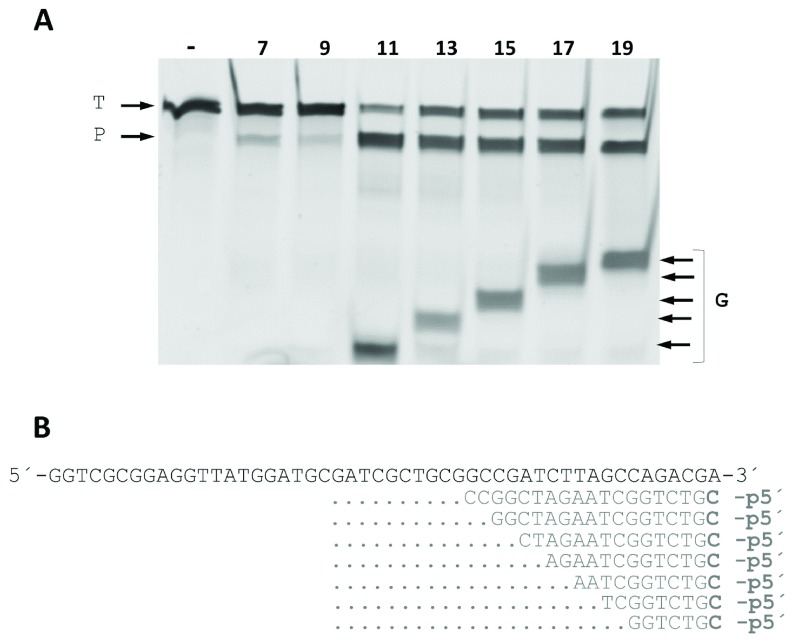
Minimum size of gDNA used by CbcAgo. ( **A**) CbcAgo was incubated with 7-, 9-, 11-, 13-, 15-, 17- or 19-mer gDNAs complementary to a ssDNA target and used them in interference cleavage assays. The target (T), guide (G) and major product (P) of the reaction were identified using an 18% U-PAGE gel. ( **B**) Sequences of the gDNAs and target ssDNA used in ( **A**).

**Figure 9.**
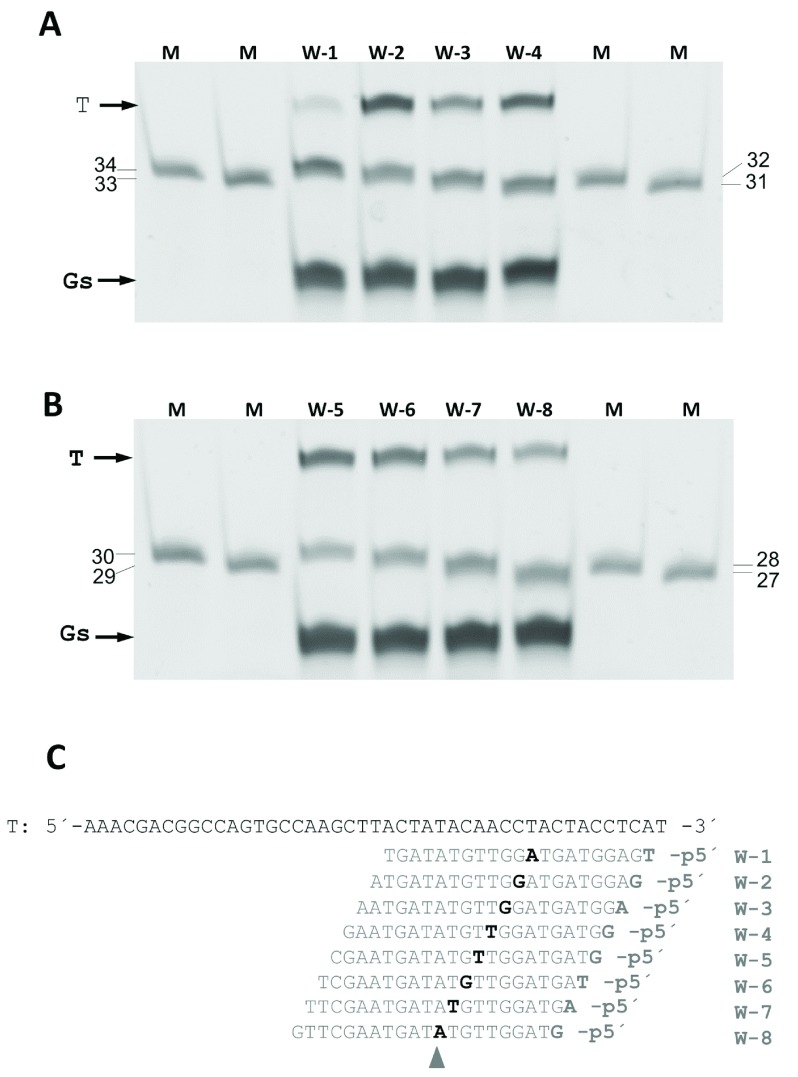
Selection of the cleavage site by CbcAgo. CbcAgo was pre-loaded with a collection of 21-mer, 5´-phosphorylated gDNA that paired at positions displaced by a single nucleotide with a 45-mer ssDNA target (T). The products of the reactions were compared using a 20% U-PAGE gel with ssDNA markers of the indicated sizes (mer). ( **A**) Assays with w1 to w4 gDNA. ( **B**) Assays with gDNA w-5 to w-8. ( **C**) Target DNA and gDNA used in **A** and **B**. Note that the cutting site was displaced along the target by a single nucleotide, always pairing at position 10–11 of the gDNAs (shaded triangle).

The minimum size of the 5’P-guides was also studied. By shortening the gDNAs at their 3’ end and using them in interference assays against the same ssDNA target, we found similar efficiencies of cleavage up to a gDNA size of 11 nucleotides ( Figure 8). Shorter gDNAs of 9- and even 7-mer still allowed the enzyme to partially cut the ssDNA target. Finally, the ability to direct the enzyme activity towards any desired site within a given ssDNA target was also demonstrated, using different guides of the same size but each with hybridization site displaced by a single base with respect to the next. Despite the fact that different efficiencies were detected, the capability of the guides to direct the wild-type CbcAgo activity against any designed site was clearly shown ( Figure 9).

**Figure 10.**
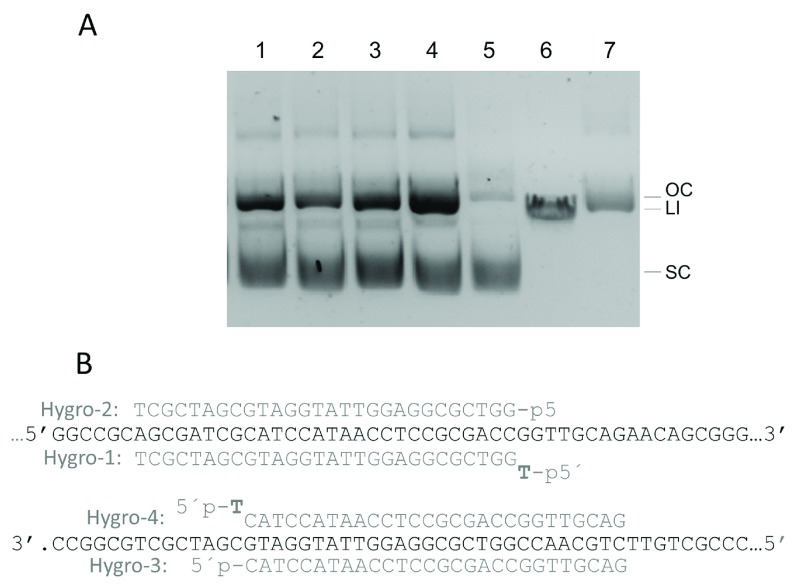
Effects of CbcAgo on dsDNA. ( **A**) CbcAgo was pre-loaded for 15 min at 37ºC with the Hygro-1 to 4 guides (lanes 1 to 4) and incubated for 16 hours at the same temperature with plasmid pMH184 that was essentially supercoiled (lane 5, SC). Linear plasmid (LI) and nicked open circle (OC) were run in lanes 6 and 7 respectively. The molar ratio between CbcAgo:guide:target was 3 μM: 6 μM: 0.0074 μM. Reactions were stopped by adding 100 μg/mL of Proteinase K (Promega) and the products separated in agarose gels. ( **B**) Sequence of the target in plasmid pMH184 paired with the gDNAs used in panel **A**.

Finally, assays on the activity of CbcAgo on dsDNA were carried out using supercoiled forms of plasmid pMH184 using four different guides complementary to the sense and antisense strands of the hygromycin resistance gene. All the gDNAs used were able to direct the production of a nick in the complementary strand, leading to the generation of open circle forms as the main product ( Figure 10). However, using a combination of primers complementary to each strand did not result in the generation of linear forms of the plasmid (not shown).

## Discussion

Novel tools based on CRISPR-Cas9 RNA-DNA interference mechanisms have been developed in the last few years, taking a leading role among the current methods for gene editing. However, significant drawbacks must be overcome in order to allow their safe use in gene therapy, especially off-target actions and putative cellular persistence of the DNA used for the editing process. The description of pAgos in thermophilic bacteria and archaea that are able to target DNA using ssDNA guides has revealed the possibility of developing a new gene editing tool, in which a mix of the protein and its synthetic gDNA would be enough to produce specific cleavage without leaving anything behind that could produce deleterious long-term effects. This possibility was apparently supported by the publication of an article claiming the use of NgAgo to modify several genes in mammalian cell cultures ^12^. However, the results were not reproducible by any of several groups ^13^. For many microbiologists, the use of a protein from a hyperhalophilic archaea was bizarre as they have evolved at the sequence level to tolerate very high potassium chloride concentrations as intracellular compatible solute. Despite this, the article sparked a growing interest in finding an appropriate pAgo protein that could have this function, as thermophilic pAgos have little or no activity at mesophilic temperatures ^11^.

In this context, the search for a mesophilic pAgo led our group (and those of J. van der Oost and A.A Aravind) to independently focus on a putative pAgo encoded by *Clostridium butyricum* due to the mesophilic character of this organism and the presence of the four domains of long pAgos and the catalytic tetrad at its PIWI domain. In a preprint article posted online, the group of J. van der Oost provided an exhaustive description of the CbAgo protein but did not identify the exact strain they used. A further work by Kuzmenko *et al.* was posted two months later with the *in vitro* characterization of the same CbAgo. In both articles, further published ^14, 15^, the same CbAgo protein was used. Although most of the findings of those articles on CbAgo were similar to the properties found for the CbcAgo protein described here, there are also some significant differences that could relate either to the differences found in the sequence of the CbAgo and CbcAgo proteins (N188D, D191G, R200K, S204A, E212K, K216N, S217T, E220D, K253N, K258Q, I343V, S466L, being the first position corresponding to the CbAgo and the second to the CbcAgo), to the presence of tags (Strep-tag in CbcAgo, His-tag in Kuzmenko´s CbAgo and no tags in Heddge`s CbAgo), or to the different experimental settings used for their characterization.

Firstly, we detected significant cleavage capacity of ssDNA targets at 55°C, whereas the CbAgo in the article of Hedgge *et al.* showed a 50°C limit for activity, a temperature at which the CbcAgo was highly active in our work, thus suggesting small thermostability differences between both proteins. However, as in the assays of the article by Kuzmenko *et al.* also with the CbAgo protein, the nuclease activity was detected even at 60°C, the apparent differences in thermostability between the three purified proteins are more likely related to the experimental settings than to the presence of amino acid changes or tags in the proteins. The main differences found between CbAgo and CbcAgo were concerning the requirement for 5´ phosphorylation of the gDNA ( Figure 4) and the minimum gDNA size required for efficient cleavage ( Figure 5). In our study, the requirement for phosphorylation of the gDNA was quite strict with CbcAgo and no nuclease activity was detected with any of the 5’-OH gDNAs used in our assays. This result is in agreement with the reported requirements of other pAgos such as ThAgo and PfAgo for gDNA phosphorylation ^3, 4^, and also with the presence of several contacts between the 5’-P end of the gDNA and specific residues revealed in the structure of CbAgo ^14^. Actually, CbAgo was less active when using 5’OH guides than when using 5’-P counterparts ^14^, and shows exceptionally high affinity to 5-P guides ^15^, supporting that the natural gDNA used *in vivo* for these pAgo are actually 5’-phosphorylated.

Even more surprising was the minimum size of gDNA required for tDNA selection and nuclease activity of CbcAgo, as gDNA of 11 nucleotides were similarly efficient in directing the CbcAgo to the correct target than longer guides, detecting also some activity for gDNAs as short as 7 nucleotides ( Figure 8). This contrasts with the minimum 12-mer required for binding of CbAgo to gDNA in single-molecule fluorescence assays and with the minimum 14-mer gDNA needed for cleavage of the ssDNA substrate reported for CbAgo ^14, 15^. The reasons for these discrepancies may be partly related to the method of detection used or to the absence of phosphorylation in some of the gDNAs used in the assays with the CbAgo protein ^14^. Whatever the case, our data for minimum gDNA requirements is more similar to the 9 nucleotides described for TtAgo ^16^.

Putting our data in the context of the structure for CbAgo described in the article published by the group of J. van der Oost ^14^, and in that of ThAgo ^6^ once even small gDNA are attached at a position of the MID domain defined by the gDNA 5’P-residue, CbcAgo is able to scan ssDNA for a matching sequence, approximating paired positions 10–11 to the active site of the PIWI domain, where the ssDNA target is cleaved in a cation-dependent manner. Smaller 5’-P- guides (i.e. 7-mers) are also efficiently recognized by the MID domain of the protein, which as described in Kuzmenko *et al.* shows a very high affinity for 5’P guides ^15^, and used to screen for complementarity. Once found (at least in an *in vitro* context without any other competing DNA) these small gDNAs could function as seed to position the tDNA near to the active site of the PIWI domain. Likely through thermodynamic movements, the tDNA eventually reach the catalytic domain in a sort of pendulum movement leading to the observed residual activity. This could be facilitated by the location of the hybridization site for the gDNA at the 3’ end of the tDNA, instead of in the middle of the target as in the assays carried out with CbAgo.

Another discrepancy has to do with the effects of CbcAgo on dsDNA. In this study, only nicking activity was detected when supercoiled plasmids were used as substrates and the corresponding gDNA was pre-bound to the protein ( Figure 10). In fact, we did not detect any chopping activity with the purified protein in the absence of gDNA, even after 16 hours of incubation at 37°C. In our assays we did not detect the linearized plasmid form, even when two gDNAs (one for each strand) were used. However, the works with CbAgo detected the double strand break on plasmids at low yields, with the activity being much more efficient in regions with low G+C content ^14, 15^, which could explain the absence of detectable linearization in a higher G+C content plasmids such as pMH184 used in our assays. This suggests that the CbcAgo protein alone is unable to open dsDNA of medium to high G+C content after its nicking activity and that future directed evolution of the protein will be needed for better adaptation to this type of substrate

## Data availability

### Underlying data

Open Science Framework: *Clostridium butyricum* CWBI1009 Ago. https://doi.org/10.17605/OSF.IO/8GQUZ ^17^

This project contains raw images of the gels used for each figure.

Data are available under the terms of the Creative Commons Zero "No rights reserved" data waiver (CC0 1.0 Public domain dedication).

## References

[1] Meister G: Argonaute proteins: functional insights and emerging roles. *Nat Rev Genet.* 2013;14(7):447–459. 10.1038/nrg3462 23732335

[2] Swarts DC Makarova K Wang Y: The evolutionary journey of Argonaute proteins. *Nat Struct Mol Biol.* 2014;21(9):743–753. 10.1038/nsmb.2879 25192263 PMC4691850

[3] Swarts DC Jore MM Westra ER: DNA-guided DNA interference by a prokaryotic Argonaute. *Nature.* 2014;507(7491):258–261. 10.1038/nature12971 24531762 PMC4697943

[4] Swarts DC Hegge JW Hinojo I: Argonaute of the archaeon *Pyrococcus furiosus* is a DNA-guided nuclease that targets cognate DNA. *Nucleic Acids Res.* 2015;43(10):5120–5129. 10.1093/nar/gkv415 25925567 PMC4446448

[5] Zander A Willkomm S Ofer S: Guide-independent DNA cleavage by archaeal Argonaute from *Methanocaldococcus jannaschii*. *Nat Microbiol.* 2017;2:17034. 10.1038/nmicrobiol.2017.34 28319081 PMC7616673

[6] Yuan YR Pei Y Ma JB: Crystal structure of A. *aeolicus argonaute*, a site-specific DNA-guided endoribonuclease, provides insights into RISC-mediated mRNA cleavage. *Mol Cell.* 2005;19(3):405–419. 10.1016/j.molcel.2005.07.011 16061186 PMC4689305

[7] Sunghyeok Y Taegeun B Kyoungmi, K: DNA-dependent RNA cleavage by the Natronobacterium gregoryi Argonaute. *bioRxiv.* 2017;1–9. 10.1101/101923

[8] Olovnikov I Chan K Sachidanandam R: Bacterial argonaute samples the transcriptome to identify foreign DNA. *Mol Cell.* 2013;51(5):594–605. 10.1016/j.molcel.2013.08.014 24034694 PMC3809076

[9] Sheng G Zhao H Wang J: Structure-based cleavage mechanism of *Thermus thermophilus* Argonaute DNA guide strand-mediated DNA target cleavage. *Proc Natl Acad Sci U S A.* 2014;111(2):652–657. 10.1073/pnas.1321032111 24374628 PMC3896195

[10] Swarts DC Szczepaniak M Sheng G: Autonomous Generation and Loading of DNA Guides by Bacterial Argonaute. *Mol Cell.* 2017;65(6):985–998 e986. 10.1016/j.molcel.2017.01.033 28262506 PMC5779613

[11] Hegge JW Swarts DC van der Oost J: Prokaryotic Argonaute proteins: novel genome-editing tools? *Nat Rev Microbiol.* 2018;16(1):5–11. 10.1038/nrmicro.2017.73 28736447

[12] Gao F Shen XZ Jiang F: DNA-guided genome editing using the *Natronobacterium gregoryi* Argonaute. *Nat Biotechnol.* 2016;34(7):768–773. 10.1038/nbt.3547 27136078

[13] Lee SH Turchiano G Ata H: Failure to detect DNA-guided genome editing using *Natronobacterium gregoryi* Argonaute. *Nat Biotechnol.* 2016;35(1):17–18. 10.1038/nbt.3753 27893702 PMC5662444

[14] Hegge JW Swarts DC Chandradoss SD: DNA-guided DNA cleavage at moderate temperatures by *Clostridium butyricum* Argonaute. *Nucleic Acids Res.* 2019;47(11):5809–5821. 10.1093/nar/gkz306 31069393 PMC6582352

[15] Kuzmenko A Yudin D Ryazansky S: Programmable DNA cleavage by Ago nucleases from mesophilic bacteria *Clostridium butyricum* and *Limnothrix rosea*. *Nucleic Acids Res.* 2019;47(11):5822–5836. 10.1093/nar/gkz379 31114878 PMC6582412

[16] Wang Y Sheng G Juranek S: Structure of the guide-strand-containing argonaute silencing complex. *Nature.* 2008;456(7219):209–213. 10.1038/nature07315 18754009 PMC4689319

[17] Berenguer J: *Clostridium butyricum* CWBI1009 Ago [Internet]. *OSF.* 2019 10.17605/OSF.IO/8GQUZ

