# Supplementary material for: DNA interference by a mesophilic Argonaute protein, CbcAgo
Source: F1000Res. 2020 Jan 9;8:321. Originally published 2019 Mar 22. [Version 2] doi: 10.12688/f1000research.18445.2 (PMC6961421; doi:10.12688/f1000research.18445.2)
Supplement: Peer review history

# Peer review: Reviewer response for version 2

**DOI:** 10.5256/f1000research.24144.r58429

**Published:** 2020-01-13

**Authors:** Nathan A. Tanner

**Affiliations:** 1 New England Biolabs, Ipswich, Massachusetts, USA

The authors addressed the major points of criticism from the original submission, with updates and additions to discuss the newer publication and discrepancies with this work. The unusual results and findings at odds with other CbAgo papers are discussed more, adding to the ability of the reader to consider this work in the field. I'd still suggest some different presentation on some of the data figures but the authors disagree and have supplemental versions with fuller images, so this can be attributed to a matter of preference. I feel the revised version is stronger and sufficient for indexing.

I confirm that I have read this submission and believe that I have an appropriate level of expertise to confirm that it is of an acceptable scientific standard.

# Peer review: Reviewer response for version 2

**DOI:** 10.5256/f1000research.24144.r58430

**Published:** 2020-01-10

**Authors:** Eduardo Santero, Inés Canosa

**Affiliations:** 1 University Pablo de Olavide, Seville, Spain; 2 Andalusian Center for Developmental Biology (CABD), Superior Council of Scientific Investigations (CSIC), Council of Andalusia, Pablo de Olavide University, Seville, Spain

I think authors have satisfactorily dealt with the reviewers comments and, therefore, I have no further comments on the revised version of the manuscript

We confirm that we have read this submission and believe that we have an appropriate level of expertise to confirm that it is of an acceptable scientific standard.

# Peer review: Reviewer response for version 1

**DOI:** 10.5256/f1000research.20180.r51940

**Published:** 2019-08-02

**Authors:** Nathan A. Tanner

**Affiliations:** 1 New England Biolabs, Ipswich, Massachusetts, USA

The study from Garcia-Quintans *et al.* looks at a new pAgo protein from Clostridium and investigates factors influencing its cleavage activity on DNA substrates. The *in vitro* activity is characterized pretty well, but there are some serious issues I find with the data and its presentation, as well as the contradictory findings of another recent publication.

1. The authors report a significant (by eye ~40%) contaminant of an N-terminal truncation at about half the size of the expected protein (Figure 1). I would assume this is an inactive form of the enzyme, but does it still bind guides? Bind to DNA targets? Perhaps affect the results of all the experiments in the paper? This should be addressed in more detail, and ideally cleaned up (along with the GroEL contaminat) using another chromatography step.
2. Most of the gels are shown as zoomed in cropped sections of the gel. I feel these should instead show the whole, or at least more of, the gel, and include low-molecular weight marker standards. Some gels have oligonucleotide standards but the resolution is very poor in terms of distinguishing between a few bases (I'd suggest moving the guides by more than 1 base). And as shown in Figure 8 11 ntd ssDNA can clearly be seen, but where is it in the other gels where the product should be 2 ssDNA's? The most problematic is Figure 5 where the far right gel is too poor for publication, and seems to show production of P species without added guide at 55C? Where is the guide in all those wells? Figure 8 seems to have additional bands between P and guide, Figure 10 has an unidentified high molecular weight species, and the size markers in Figure 7 should be labeled more clearly. 
3. I feel there should be more explanation given to the (to me) bizarre finding that a 7 or 9 base guide can cut at the +10/11 position...which of course does not have a guided complement. How do the authors think this can happen?
4. The authors mention the Hegge *et al.*, preprint, which they should, but that paper was published in NAR after this study. And importantly, so was another study with CbAgo, from a strain mentioned here (Kuzmenko *et al.* ^1^). In this study, the authors show several things at odds with the current work: no cleavage with 10 or 12-base guides even after 24hr incubation, activity to 60C, ability to use 5'-OH guides, the ability to cut dsDNA with opposite strand guides at 37C in 1-4h, and with moderate (500 nM) concentrations of CbAgo a chopping activity on plasmid DNA. It is likely this work was not available at the time the reviewed study was published, but it is difficult to ignore the contradictions now. It is possible that the Cb/CbcAgo protein is exactly the same in all 3 studies, and these discrepancies are significant for the conclusions presented here.
5. Related, I'd expect there to be some plasmid chopping given the time and concentrations the authors describe. But no Apo reactions are shown in Figure 10, an important control that is left out. And a comparison of attempts to digest non-supercoiled plasmid would be good for the explanation that dsDNA cannot be accessed w/o supercoiling.
6. Minor points, but there are some errors ("xilencyanol", "ImajeJ) and inconsistencies (PfAgo/PfuAgo) that should be fixed.

I confirm that I have read this submission and believe that I have an appropriate level of expertise to confirm that it is of an acceptable scientific standard, however I have significant reservations, as outlined above.

## References

[1] : Programmable DNA cleavage by Ago nucleases from mesophilic bacteria Clostridium butyricum and Limnothrix rosea. *Nucleic Acids Res*.2019;47(11) : 10.1093/nar/gkz379 5822-5836 10.1093/nar/gkz379 31114878PMC6582412

# Response

**Published:** 2019-12-23

**Authors:** Jose Berenguer

**Affiliations:** Universidad Autónoma de Madrid, Spain

**Reviewer  2**

 García-Quintans et al. reports on the identification of an Argonaute protein coding gene from the mesophilic bacterium *Clostridium butyricum* strain CWBI 1009, and characterise its product as a potential genome editing tool alternative to the well-known CRISPR/Cas. Authors have purified the native protein and an inactive variant as a control, and thoroughly characterise its activity in relation to a number of parameters such as different temperatures, cations and ionic strength. Altogether, the work of García-Quintans is a well-designed characterisation of the in vitro activity of the CbcAgo protein.

 A similar work has been recently published in Nucleic Acids Research1, by the group of John van der Oost using a similar CbAgo protein from an unspecified strain of C. butyricum, as acknowledged by the authors. It appears that both proteins have very similar characteristics.

 The authors claim some relevant differences between both proteins:

**Enzyme stability at different temperatures:** In first place, none of authors assay thermostability, they assay activity at different temperatures, which is not the same. This should be changed in the Discussion, pg. 10. Based on the partial activity detected at 55°C in this paper, authors claim CbcAgo might be more “stable” than CbAgo (Discussion, pg. 10). However, CbAgo activity was not assayed at 55°C but at 50°C (partially active) and 64°C (inactive). A comparison of the activities at the same temperature, 50°C, which show almost maximal activity of CbcAgo but substantially reduced activity of CbAgo (<50%), would be more reliable. Anyways, since the difference is very small, it is very difficult to ascertain whether the differences are real or a consequence of slightly different assay conditions.

*Answer:*

*In a further article by Kuzmenko et all, the CbAgo protein shows nuclease activity up to 60ºC, so the apparent thermostability differences are more likely due to the experimental settings used than to the protein sequence itself. We have modified the discussion accordingly just saying that the apparent activity at high temperature is higher under our experimental conditions, but that due to differences in the assays (dDNA and tDNA) the data cannot be directly compared*

**Strict dependence on phosphorylation:** Both CbAgo and CbcAgo, were unable to cut a short 45-mer target DNA if the gDNA is 5’-OH. However, Hegge et al., 20191, additionally reported partial activity of CbAgo on a longer target (120-mer), which was not tested in this manuscript. Therefore, in this regard, there is no data that supports the difference between both proteins claimed in this manuscript.

*Answer:*

*This has been already commented as answer to point 4 raised by reviewer  1. We agree in that even for the CbAgo it is more likely to use a 5’P gDNA than an 5’-OH one, as i) they are less active in the best of the cases than  5’-P counterparts, and ii) the binding site at the MID domain shows several contacts between the 5’P extreme of the gDNA and residues in the protein, supporting a higher affinity for this type of substrates than for  5’OH ones.  The differences found are more likely due to differences in the experimental setting, including the selection of the substrates and the hybridization position.*

**Minimum size length of the gDNA:** By comparing the results obtained with both proteins, it is apparent that CbcArgo requires a shorter gDNA to cleave the target. I think this is the most evident difference. However, as the authors acknowledge, these differences may be due to technical reasons rather than a difference in catalytic activity between the Argo proteins. The question of whether CbcArgo and CbArgo show any difference in activity could only be solved by making a side by side comparison of both proteins having the same tag, and using exactly the same procedure.

 Anyways, I really miss an alignment of CbAgo and CbcAgo proteins to know how different these proteins are at the amino acid level.

*Answer:*

*Proteins CbAgo (used in the articles of Hedgge et al and Kuzmenko et al and CbcAgo (Used in this article) are very similar. From the 748 amino acids of the three protein only 12 differences exist: N188D, D191G, R200K, S204A, E212K, K216N, S217T, E220D, K253N, K258Q, I343V, S466L, being the first position corresponding to the CbAgo and the second to the CbcAgo.  These changes  are located mainly at inter-domain  regions and could be not too relevant for the activity but could affect parameters such as stability.  Additional differences in the results between both proteins could be due to the presence or absence of tags (Strep-tag in CbcAgo, His-tag in Kuzmenko´s  CbAgo and no tags in Heddge`s CbAgo).*

 Other comments:

 Please, properly align lanes and lanes names/numbers in Fig. 1.

*Answer:*

*OK, done*

 Requirement for 5´phosphorylated gDNA is shown in Fig. 6, not 4 (Discussion, pg. 10).

*Answer:*

*OK, Modified*

*Answer:*

*Actualized references included in the text:*

*Hegge, J. W. et al. DNA-guided DNA cleavage at moderate temperatures by Clostridium butyricum Argonaute. Nucleic Acids Res.* *47, 5809-5821. doi: 10.1093/nar/gkz306.(2019).*

*Kuzmenko, A.,  Yudin, D.,  Ryazansky, Kulbachinskiy, S. A. & Aravin, A. A.  Programmable DNA cleavage by Ago nucleases from mesophilic bacteria Clostridium butyricum and Limnothrix rose. Nucleic Acids Res. **47**, 5822–5836 doi: 10.1093/nar/gkz379 (2019)*

# Peer review: Reviewer response for version 1

**DOI:** 10.5256/f1000research.20180.r51304

**Published:** 2019-07-30

**Authors:** Eduardo Santero, Inés Canosa

**Affiliations:** 1 Andalusian Center for Developmental Biology (CABD), Superior Council of Scientific Investigations (CSIC), Council of Andalusia, Pablo de Olavide University, Seville, Spain; 2 University Pablo de Olavide, Seville, Spain

García-Quintans *et al.* reports on the identification of an Argonaute protein coding gene from the mesophilic bacterium *Clostridium butyricum* strain CWBI 1009, and characterise its product as a potential genome editing tool alternative to the well known CRISPR/Cas. Authors have purified the native protein and an inactive variant as a control, and thoroughly characterise its activity in relation to a number of parameters such as different temperatures, cations and ionic strength. Altogether, the work of García-Quintans is a well-designed characterisation of the in vitro activity of the CbcAgo protein.

 A similar work has been recently published in Nucleic Acids Research ^1^, by the group of John van der Oost using a similar CbAgo protein from an unspecified strain of *C. butyricum*, as acknowledged by the authors. It appears that both proteins have very similar characteristics.

 The authors claim some relevant differences between both proteins:

**Enzyme stability at different temperatures:** In first place, none of authors assay thermostability, they assay activity at different temperatures, which is not the same. This should be changed in the Discussion, pg. 10. Based on the partial activity detected at 55°C in this paper, authors claim CbcAgo might be more “stable” than CbAgo (Discussion, pg. 10). However, CbAgo activity was not assayed at 55°C but at 50°C (partially active) and 64°C (inactive). A comparison of the activities at the same temperature, 50°C, which show almost maximal activity of CbcAgo but substantially reduced activity of CbAgo (<50%), would be more reliable. Anyways, since the difference is very small, it is very difficult to ascertain whether the differences are real or a consequence of slightly different assay conditions.

**Strict dependence on phosphorylation:** Both CbAgo and CbcAgo, were unable to cut a short 45-mer target DNA if the gDNA is 5’-OH. However, Hegge *et al.*, 2019 ^1^, additionally reported partial activity of CbAgo on a longer target (120-mer), which was not tested in this manuscript. Therefore, in this regard, there is no data that supports the difference between both proteins claimed in this manuscript.

**Minimum size length of the gDNA:** By comparing the results obtained with both proteins, it is apparent that CbcArgo requires a shorter gDNA to cleave the target. I think this is the most evident difference. However, as the authors acknowledge, these differences may be due to technical reasons rather than a difference in catalytic activity between the Argo proteins. The question of whether CbcArgo and CbArgo show any difference in activity could only be solved by making a side by side comparison of both proteins having the same tag, and using exactly the same procedure.

 Anyways, I really miss an alignment of CbArgo and CbcArgo proteins to know how different these proteins are at the amino acid level.

**Other comments:**

 Please, properly align lanes and lanes names/numbers in Fig. 1.

 Requirement for 5´phosphorylated gDNA is shown in Fig. 6, not 4 (Discussion, pg. 10).

We confirm that we have read this submission and believe that we have an appropriate level of expertise to confirm that it is of an acceptable scientific standard, however we have significant reservations, as outlined above.

## References

[1] : DNA-guided DNA cleavage at moderate temperatures by Clostridium butyricum Argonaute. *Nucleic Acids Res*.2019;47(11) : 10.1093/nar/gkz306 5809-5821 10.1093/nar/gkz306 31069393PMC6582352

# Response

**Published:** 2019-12-23

**Authors:** Jose Berenguer

**Affiliations:** Universidad Autónoma de Madrid, Spain

**Reviewer 1.**

 The study from Garcia-Quintans et al. looks at a new pAgo protein from Clostridium and investigates factors influencing its cleavage activity on DNA substrates. The in vitro activity is characterized pretty well, but there are some serious issues I find with the data and its presentation, as well as the contradictory findings of another recent publication.

 1.            The authors report a significant (by eye ~40%) contaminant of an N-terminal truncation at about half the size of the expected protein (Figure 1). I would assume this is an inactive form of the enzyme, but does it still bind guides? Bind to DNA targets? Perhaps affect the results of all the experiments in the paper? This should be addressed in more detail, and ideally cleaned up (along with the GroEL contaminat) using another chromatography step.

*Answer:*

*As described in the article these contaminant proteins were systematically associated to the full length protein. The use of an inactive mutant cbAgo in which the N-terminal truncated fragment and the GroEL proteins were also co-purified guarantees that the observed activity on the target DNA in the presence of gDNA is exclusively due to the catalytic activity of the wt enzyme and not the result of hidden activity of the co-purified proteins. A series of different chromatographic steps have been tried, and in all cases separation of contaminants was not possible and/or the activity of the enzyme was diminished*

*However we agree in that N-terminal fragment could bind either target or gDNA, leading to a minor subestimation of the actual activity of the enzyme or the optimal ratio target:gDNA, but without affecting the conclusions shown in the article.*

 2.            Most of the gels are shown as zoomed in cropped sections of the gel. I feel these should instead show the whole, or at least more of, the gel, and include low-molecular weight marker standards. Some gels have oligonucleotide standards but the resolution is very poor in terms of distinguishing between a few bases (I'd suggest moving the guides by more than 1 base). And as shown in Figure 8 11 ntd ssDNA can clearly be seen, but where is it in the other gels where the product should be 2 ssDNA's? The most problematic is Figure 5 where the far right gel is too poor for publication, and seems to show production of P species without added guide at 55C? Where is the guide in all those wells? Figure 8 seems to have additional bands between P and guide, Figure 10 has an unidentified high molecular weight species, and the size markers in Figure 7 should be labeled more clearly.

*Answer:*

*Due to requirements for organizing easy-to-follow figures, the images are cropped, as most figures in articles in the field are. However, as described in the article, the original full size images of all the gels used are accessible for detailed analysis at https://doi.org/10.17605/OSF.IO/8GQUZ*

*Regarding gel resolution, we do not agree with the reviewer. Under the conditions used a single base makes a difference in the detected mobility of the gDNA and products, and our interest of using base-to –base changes was to be sure that we were able to move the cutting site in the tDNA in a precise manner (i.e. figs 7,  9). As there are so many size markers, only those closer to the size of the products were indicated, but likely the size of the numbers is too small for readers and are increased in the  revised version.*

*Regarding Fig 8, as the reviewer comments the reaction generates two ssDNA products from the tDNA.  The  largest one is 40 mer and is detected and  labelled as P in the figures, and this is also the most adequate fragment for comparison. The small one is only 10 mer long (at the 3’ extreme of the tDNA) and escapes from the standard gels (lane 11 in Fig 8 shows the 11mer gDNA at the bottom of the gel). Also, in the other figures the small tDNA product moves out of the gels due to its small size.*

*In figure 8, the reviewer points to the presence of minor bands below the main 40 mer product, especially under the lanes labelled 11 and 13.  We cannot be sure of their origin but could correspond to lower affinity matches of the guides (11 and 13 mer) with the tDNA, as they are not detected with longer guides.*

*Regarding figure 5, the reviewer is right. The line indicating absence (-) or presence (+) of guides have contracted during the figure assembly and it apparently seems that the enzyme cuts without guide at 50 and 55 ºC, but it is not that way.  In the gel at right,  the left lanes below each temperature (55,  60, 65 and 70 ºC) corresponds to assays without gDNA and the right one with gDNA (detected at the gel bottom), whereas in the  left and central panels is first with (+) and then without (-) .  In all cases, the product is only detected when the guide is present.  This figure will be modified to correct the alignment of the labellings.*

*Respect to Fig. 10, the high molecular size  species detected could be concatenated plasmids, as it was detected in the untreated plasmid preparation (lane 5) and in the open circle treatment  (lane 7) but not in the linear form (lane 6).*

 3.            I feel there should be more explanation given to the (to me) bizarre finding that a 7 or 9 base guide can cut at the +10/11 position...which of course does not have a guided complement. How do the authors think this can happen?

*Answer:*

*As the reviewer comments the ability of CbAgo to cut with low efficiency tDNA using guides so small that their 3’ end do not reach the catalytic residue  in the protein where the cut is carried out was  a surprising  feature ( at least  11mer pairing would be needed as deduced from the structure in Hedge et al).   Under our experimental conditions 5’-phosphorilated guides of 11 or more nucleotides are very efficient triggering the enzyme activity at complementary 10/11 position of the tDNA (Fig. 8).  Smaller P-guides of 9 and even of 7 mer are still able to direct the enzyme to the expected cutting site, but with much lesser efficiency (Fig  8).  The explanation that we find for this is that the small 5’-P- guides are also efficiently recognized by the MID domain of the protein and used to screen for complementarity. Once found (in a context without any other competing DNA) these small gDNAs could function as seed to position the tDNA  near to the active site of the PIWI domain. Likely through thermodynamic movements, the target DNA eventually reach the catalytic domain in a sort of pendulum movement leading to the observed residual activity.  This explanation has been included in the text for clarification*

 4.            The authors mention the Hegge at al., preprint, which they should, but that paper was published in NAR after this study. And importantly, so was another study with CbAgo, from a strain mentioned here (Kuzmenko et al). In this study, the authors show several things at odds with the current work: no cleavage with 10 or 12-base guides even after 24hr incubation, activity to 60C, ability to use 5'-OH guides, the ability to cut dsDNA with opposite strand guides at 37C in 1-4h, and with moderate (500 nM) concentrations of CbAgo a chopping activity on plasmid DNA. It is likely this work was not available at the time the reviewed study was published, but it is difficult to ignore the contradictions now. It is possible that the Cb/CbcAgo protein is exactly the same in all 3 studies, and these discrepancies are significant for the conclusions presented here.

*Answer:*

*As the reviewer indicates the article preprint by Kuzmenko was not available when the present article was sent for publication. The revised version includes the actual publication of both articles by Hegge et al and Kuzmenko et al in Nucleic Acids Research, both dealing with the same version of the CbAgo protein.*

*The main discrepancies between our work and those mentioned above  (which also show discrepancies between them) are the minimum size of the guides required for efficient cutting, smaller in our work,  and the requirement for 5’-phosphorilation of our guides for efficient activity, and both are likely related.*

*Phosphorilation of the guides:  In the referred articles the use of 5’OH-gDNA under their experimental conditions allows the cutting of tDNA by CbAgo at lower efficiency than with phosphorylated guides (actually  suggesting that the natural function for these proteins could require phosphorylated guides), whereas under our experimental conditions the CbcAgo protein was basically inactive at using 5’ OH-guides  of 20 and 21 mer compared to the phosphorylated ones.  The reasons underlying this difference could be related  differnte and likely concurrent experimental differences. First, our gDNA hibridizes at the 3’ extreme of the tDNA instead of acting in the middle of the target as in Kuzmenko et al.  Second,  the size of the tDNA could also have an effect, as suggested by  supplementary figure 4 of the article by Hegge et al in which a 5’OH guide does not cut a 45 mer target (identical in size to that used in our experiments), whereas it functions with a tDNA of 120 mer. Third, differences in the sequence of the CbAgo (used in Heddge and Kuzmenco works) and CbcAgo proteins (12 amino acid positions) or in their affinity taggings (Strep-tag in our work, His-tag in Kuzmenko´s and no tags in Heddge) could have effects on their performance. In any case, as commented above preference for 5’P guides for CbcAgo is clear in all the cases, and could be significant regarding its actual function in a cell context.*

*Minimum size of the guides:  The preference for 5’P guides by CbcAgo under our experimental conditions  could also be the basis for  experimental discrepancies regarding the minimum guide size to direct the protein, as in our experiments all our guides were phosphorilated, whereas in the works of Heddge et al the guides are labelled with a large fluorophore (Cy3), and in that of Kuzmenko et al. the guides hybridize in the middle of larger tDNA instead of pairing starting at the 3’ extreme of the tDNAs as we did in our experimental setting.*

*DNA chopping: Another point raised by the reviewer focus on the so called “chopping” activity of the enzyme on double stranded DNA. In our hands, we did not observe any degradative activity on the plasmid used as target  (pMH184) even  after 16 hours of incubation at 37ºC with significant concentrations of CbAgo protein loaded with gDNA (Fig 10) or without gDNA  (experiment not shown).*

*dsDNA activity: Regarding cutting of supercoiled plasmid, it seems clear that nicks were generated using CbcAgo loaded with guides directed to both DNA strands of a supercoiled plasmid, but under our experimental condition we did not detect linearized plasmid, supporting interference between overlapping cutting sites*

 5.            Related, I'd expect there to be some plasmid chopping given the time and concentrations the authors describe. But no Apo reactions are shown in Figure 10, an important control that is left out. And a comparison of attempts to digest non-supercoiled plasmid would be good for the explanation that dsDNA cannot be accessed w/o supercoiling.

*Answer:*

*As commented above, no chopping was detected in our experiments despite incubation for 16 h at 37ºC with the protein in nmolar ratios 10:1 with the dsDNA plasmid*

 6.            Minor points, but there are some errors ("xilencyanol", "ImajeJ) and inconsistencies (PfAgo/PfuAgo) that should be fixed.

*Answer:*

*Corrected in the revised form*
